# Picoliter Well Array Chip-Based Digital Recombinase Polymerase Amplification for Absolute Quantification of Nucleic Acids

**DOI:** 10.1371/journal.pone.0153359

**Published:** 2016-04-13

**Authors:** Zhao Li, Yong Liu, Qingquan Wei, Yuanjie Liu, Wenwen Liu, Xuelian Zhang, Yude Yu

**Affiliations:** 1 State Key Laboratory on Integrated Optoelectronics, Institute of Semiconductors, Chinese Academy of Sciences, P.O. Box 912, Beijing, 100083, China; 2 Joint Laboratory of Bioinformation Acquisition and Sensing Technology, Institute of Semiconductors, Beijing Institute of Genomics, Chinese Academy of Sciences, P.O. Box 912, Beijing 100083, China; Texas A&M University, UNITED STATES

## Abstract

Absolute, precise quantification methods expand the scope of nucleic acids research and have many practical applications. Digital polymerase chain reaction (dPCR) is a powerful method for nucleic acid detection and absolute quantification. However, it requires thermal cycling and accurate temperature control, which are difficult in resource-limited conditions. Accordingly, isothermal methods, such as recombinase polymerase amplification (RPA), are more attractive. We developed a picoliter well array (PWA) chip with 27,000 consistently sized picoliter reactions (314 pL) for isothermal DNA quantification using digital RPA (dRPA) at 39°C. Sample loading using a scraping liquid blade was simple, fast, and required small reagent volumes (i.e., <20 μL). Passivating the chip surface using a methoxy-PEG-silane agent effectively eliminated cross-contamination during dRPA. Our creative optical design enabled wide-field fluorescence imaging in situ and both end-point and real-time analyses of picoliter wells in a 6-cm^2^ area. It was not necessary to use scan shooting and stitch serial small images together. Using this method, we quantified serial dilutions of a *Listeria monocytogenes* gDNA stock solution from 9 × 10^-1^ to 4 × 10^-3^ copies per well with an average error of less than 11% (N = 15). Overall dRPA-on-chip processing required less than 30 min, which was a 4-fold decrease compared to dPCR, requiring approximately 2 h. dRPA on the PWA chip provides a simple and highly sensitive method to quantify nucleic acids without thermal cycling or precise micropump/microvalve control. It has applications in fast field analysis and critical clinical diagnostics under resource-limited settings.

## Introduction

Digital polymerase chain reaction (dPCR) is a powerful method for nucleic acid detection and absolute quantification, in which the diluted sample and reaction components are partitioned into hundreds, or even millions, of individual, parallel reaction chambers so that each contains one or no copy of the templates [1–4]. After endpoint amplification, the template concentration in the original sample is determined by a Poisson statistical analysis of the number of “positive” partitions (in which the amplified target is detected) versus “negative” partitions (in which it is not). dPCR offers many advantages over quantitative PCR (qPCR) [5], such as absolute quantification without dependence on cycle thresholds or external references, and much higher accuracy and sensitivity [6–9]. Applications of dPCR span many areas of biology, including liquid biopsy [10–12], copy number variation analysis [13,14], rare sequence detection [15,16], gene expression analysis [5,17,18], single-cell genomics analysis [19–21], pathogen detection and microbiome analysis [2,22,23], as well as calibration for next-generation sequencing [24].

dPCR has been successfully performed in a variety of formats, such as multiwell plates [1,3], an emulsion PCR [25–27], microdroplets [19,28–31], microfluidic chambers [32], a spinning disk platform [33], and a SlipChip [34,35], and these can be summarized as chip-based or droplet-based dPCR [4]. However, most of the reported microfluidic chips still require a complex fabrication process, many tubes for liquid transportation, syringe pumps for pressure-driven flow, and a pneumatic system for microvalve control [36]. Current droplet-based approaches also require pumping equipment and an external rapid readout device. To ensure a homogeneous droplet size distribution, the flow rate of droplet production by T-junctions [37,38] or flow focusing [39–41] must be precisely controlled. Moreover, all of these dPCR methods still require thermal cycling and accurate temperature control.

To avoid thermal cycling, different isothermal amplification methods have been developed that rapidly amplify nucleic acids to detectable levels at a single temperature [42,43], such as loop-mediated amplification (LAMP) [44], rolling circle amplification (RCA) [45], helicase-dependent amplification (HDA) [46], nucleic acid sequence-based amplification (NASBA) [47], recombinase polymerase amplification (RPA) [48], transcription-mediated amplification (TMA) [49], multiple displacement amplification (MDA) [50], and strand-displacement amplification (SDA) [51]. In contrast to other isothermal amplification techniques, RPA offers several important advantages for point-of-care applications: it requires a lower amplification temperature of between 25°C and 42°C, is tolerant to impure samples, amplifies targets to detectable levels more rapidly (15–30 min), and uses lyophilized enzymes without cold chain storage and transport [48].

Recently, a number of reports have proposed RPA-based strategies for pathogen detection. The detection formats include fluorescence detection in real time or endpoint detection via a lateral flow strip [52–55]. Reverse transcription RPA can be also used to detect RNA targets from foot-and-mouth disease virus or Middle East respiratory syndrome coronavirus [56,57]. Additionally, RPA is highly sensitive for the detection of HIV proviral DNA and *Mycobacterium tuberculosis* DNA [52,55,58]. Instrument-free and electricity-free RPA can be performed using body heat or heating achieved using sodium acetate trihydrate for nucleic acid diagnostic tests in low-resource settings that lack expensive thermal cycling PCR equipment or even electric power [59,60].

In contrast to the common dPCR, the developments of digital isothermal amplification techniques are still insufficient. To the best of our knowledge, only LAMP, MDA, SDA, RCA, and RPA have their corresponding digital amplification techniques, which are almost performed in droplets or microfluidic chips [61–65]. Digital RPA (dRPA) has been implemented only on the SlipChip and in droplets produced by centrifugal step emulsification [66,67]. The method requires extensive development to enable applications to other simple platforms, without any complicated and precise control.

In this work, we developed a picoliter well array (PWA) chip to perform real-time dRPA for absolute quantification of nucleic acids. The PWA chip was manufactured from a silicon substrate and etched with 27,000 consistently sized picoliter wells. We demonstrated that sample loading using a scraping liquid blade is suitable for the PWA chip, which was simpler, faster, and resulted in less reaction dropout than droplet-based approaches. Additionally, unlike other microfluidic chips, it did not require a complex fabrication process or a pneumatic control system. Passivating the PWA chip surface using a methoxy-PEG-silane agent eliminated cross-contamination. For wide-field fluorescence imaging in situ, we creatively placed an optical cube between the chip and object lens to reduce the loss of light intensity in a conventional microscope. Not only did this ensure that the light intensity was able to excite the fluorescence signal, but it also avoided the need to obtain images by scan shooting and stitching serial small images together. Finally, we sealed the PWA chip in a homemade copper chamber filled with oil and successfully performed real-time dRPA on an isothermal incubation setup for the absolute quantification of serial dilutions of a *Listeria monocytogenes* gDNA stock solution.

## Materials and Methods

### Fabrication and silanization of the PWA chip

The substrate for the PWA chip was an n-type (100) single crystalline silicon wafer, with a thickness of 500 μm and a diameter of 100 mm. The silicon wafer was chemically cleaned by sequential immersion in acetone, ethanol, and deionized (DI) water for 15 min, with sonication. Then, it was immersed in a Piranha solution (H_2_SO_4_:H_2_O_2_ = 3:1) at 180°C for 30 min, rinsed thoroughly with DI water, and dried with nitrogen. Positive photoresist AZ 6130 (thickness, 5 μm) was spin-coated on the silicon wafer for subsequent lithography (MA6; SUSS MicroTec, Garching, Germany) with a photolithography mask. Under the protection of the photoresist, a picoliter well array was formed using deep reactive-ion etching (PlasmaLab System 100; Oxford Instruments, Concord, MA, USA) and the remaining photoresist was removed with acetone. After cleaning, a 200-nm-thick silicon dioxide (SiO_2_) layer was added to the wafer in a thermal oxidation furnace at 1050°C.

To covalently couple methoxy-PEG-silane (2-[methoxy(polyethyleneoxy)propyl]-trimethoxysilane; J&K Scientific, Beijing, China) to the PWA chip surface, the bare chips were treated in an oxygen plasma for 30 s to clean the surfaces (Mercator Control Systems, Inc., Santa Ana, CA, USA). Then, they were placed in 1 g of methoxy-PEG-silane dissolved in 100 mL of anhydrous toluene with 1% triethylamine as a catalyst overnight at room temperature [68,69]. Before the application of the PWA chip, the physically adsorbed methoxy-PEG-silane moieties were removed by rinsing the chip in ethanol and DI water, sequentially. Finally, the PWA chip was dried with nitrogen and used immediately.

### Preparation of dRPA reagents

All RPA reactions were performed using the RPA Exo Kit (TwistDx, Cambridge, UK) according to the manufacturer's protocol, with the exception of the addition of Mg^2+^. After Mg^2+^ was added, amplification began, unlike conventional PCR triggered by a “hot-start.” Genomic DNA from *L*. *monocytogenes* strain EGDe (ATCC, Manassas, VA, USA) gDNA was used at concentrations given for the dRPA. Dilution was performed in sterile tubes using DNase/RNase-free water (Thermo Fisher Scientific, Waltham, MA, USA). The primers and probes were provided in the *L*. *monocytogenes* Kit from TwistDx. The sequences can be found in S1 Table. To prepare 50-μL reactions, 5 μL of resuspended oligo mix, 29.5 μL of TwistAmp rehydration buffer, and 11.5 μL of DNA sample were vortexed and centrifuged briefly in a 1.5-mL tube. Subsequently, the reaction mix was used to rehydrate the freeze-dried enzyme pellet. To avoid amplification in the bulk, 4 μL of 280 mM MgAc solution was prepared separately on ice and added to the ice-cold reaction mixture (oligo mix, buffer, template, and enzyme) directly prior to fast sample loading.

The *L*. *monocytogenes* gDNA stock solution was prepared by rehydrating the dried DNA powder with DNase/RNase-free water. The final concentration was 40 pg μL^−1^, which was verified using a NanoDrop spectrophotometer (Thermo Fisher Scientific). One picogram of *L*. *monocytogenes* gDNA contained approximately 315 copies. The volume of reaction solution in each microwell was 314 pL, and for a gDNA sample concentration of 40 pg μL^-1^, approximately 9 × 10^-1^ copies per well was expected. The detailed description of the calculating method is shown in S1 Fig.

### Sample loading and chip packaging

**Fig 1:**
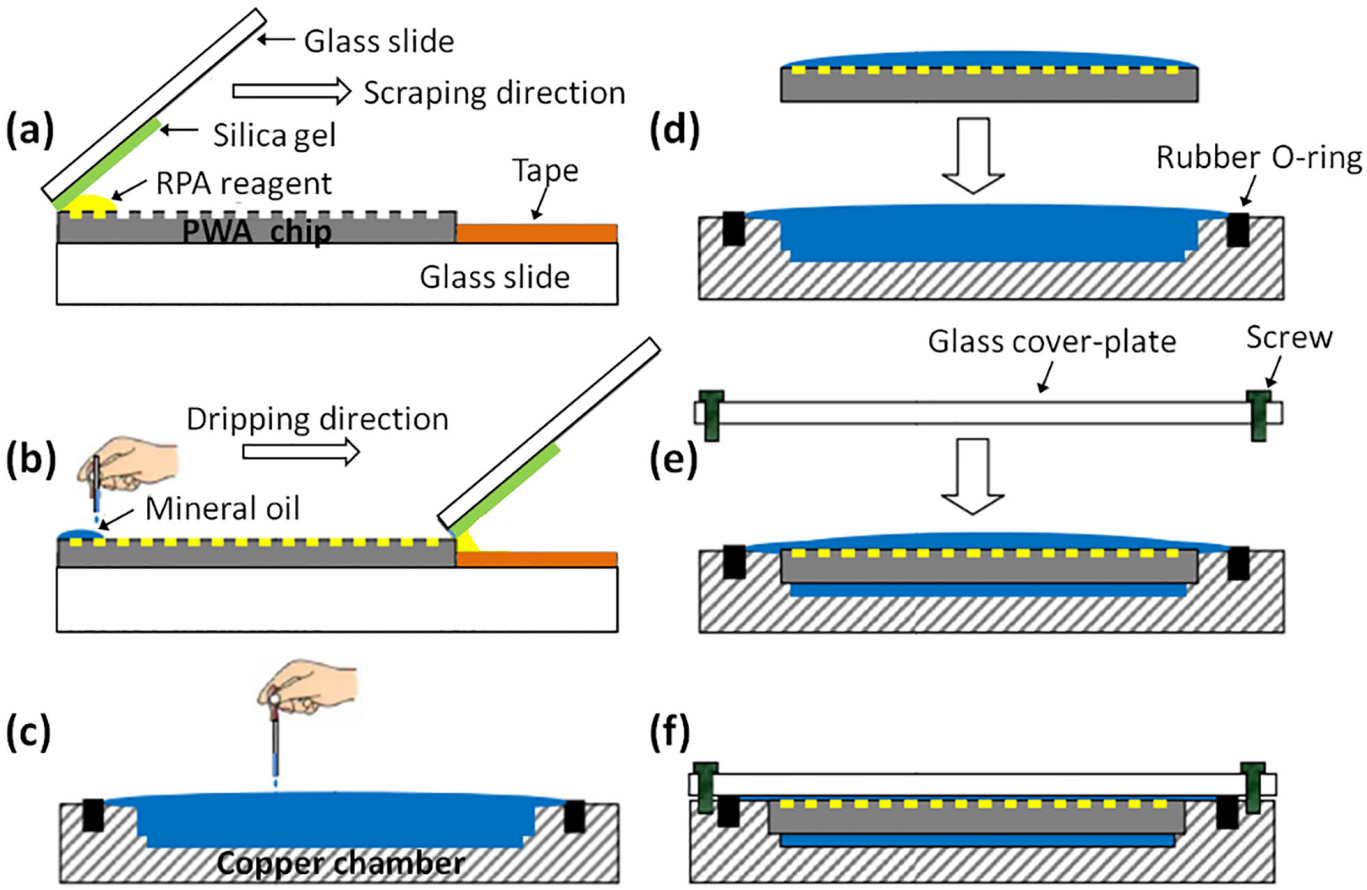
Workflow of sample loading and chip packaging. (a) The sample loading instrument. (b) Scraping reagents into picoliter wells and overlaying the chip with excess mineral oil. (c—d) Filling the copper chamber with mineral oil prior to transferring and packaging the finished PWA chip. (e—f) Fixing a glass cover-plate on the copper chamber with screws.

The sample loading instrument included a scraping liquid blade and a chip carrier. Detailed operation steps are shown in Fig 1. The scraping liquid blade was composed of a hard glass slide and a piece of soft silica gel (thickness, 3 mm), which were pasted together at an end; the chip carrier was composed of another glass slide and a 3M adhesive tape, which prevented the chip from sliding in the process of scraping (Fig 1a). The prepared 20-μL digital RPA reagents were carefully transferred onto one end of the PWA chip by a pipette. Depressing the pipette to the second stop was unsuitable for reducing the introduction of air bubbles to the chip. The blade was held at a 40–60° angle relative to the chip carrier so that the edge of silica gel was placed at the end of PWA chip. The angle of the blade was adjusted slightly until visual confirmation that RPA reagents were wetting the chip. Then, in one smooth motion, the blade was dragged slowly across the chip, while a slight downward pressure was applied to dispense the reagent (Fig 1a and 1b). The scraping time was approximately 5 s. The PWA chip sat at room temperature for 20 s for residual liquid evaporation. The chip was then gently overlaid with excess mineral oil (M8410; Sigma-Aldrich, St. Louis, MO, USA) via a disposable pipette until the entire surface was fully covered (Fig 1b). Mineral oil was used because it is lighter than water (0.82–0.88 g mL^−1^) and its solubility in water is extremely low [9]. It makes the RPA reaction independent of each other. Therefore, the subsequent packaging operation can not affect the accuracy of dRPA results, even at room temperature. The copper chamber was filled with mineral oil prior to transferring and packaging the finished PWA chip after sample loading (Fig 1c and 1d). Finally, a piece of 2.5-mm-thick quartz glass cover-plate was fixed on the copper chamber by four screws (Fig 1e and 1f). The whole process avoided air bubbles. To strengthen the air tightness between the glass and the copper chamber, a rubber O-ring was added around the chamber. The bright images of the sample loading and chip packaging process can be found in S2 Fig.

### Isothermal incubation setup for dRPA-on-chip

**Fig 2:**
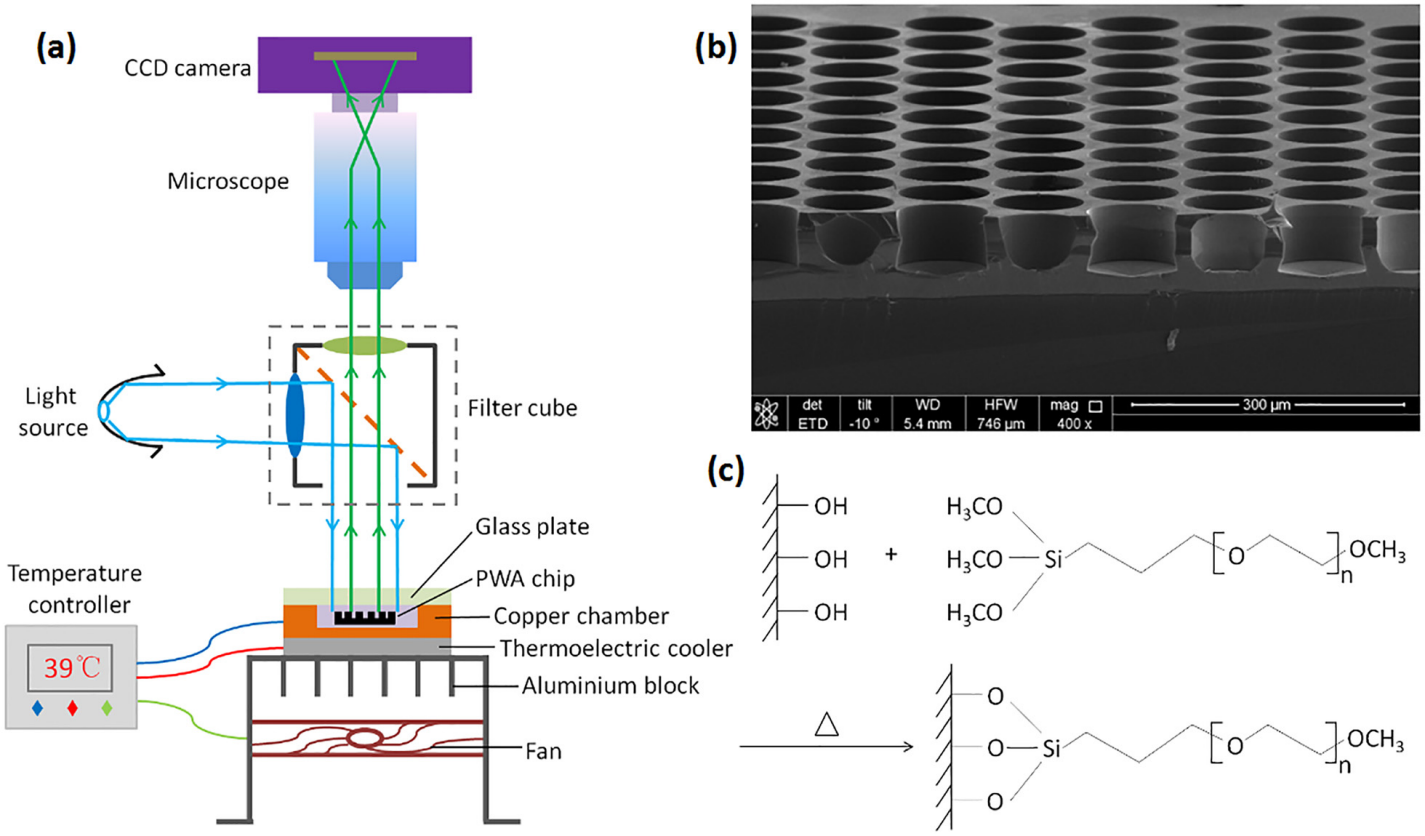
(a) Isothermal incubation setup and wide-field fluorescence imaging setup. (b) Scanning electron microscope (SEM) image of the picoliter well array. Scale bar represents 300 μm. (c) Passivating the PWA chip surface by covalently coupling methoxy-PEG-silane.

The isothermal incubation setup for dRPA-on-chip was achieved using a 40 mm × 40 mm thermoelectric cooler (TEC), which was controlled by a PID temperature controller AI-518 (Yudian Automation Technology, Xiamen, China) with LabVIEW software (National Instruments, Austin, TX, USA) (Fig 2a). Temperature feedback was accomplished by inserting a 1-mm-thick PT100 thermistor between the chip packaging device and the TEC unit. A 12 V × 3 A fan and a custom-fabricated aluminium block were placed beneath the TEC unit to dissipate waste heat. After sample loading and chip sealing, the picoliter droplets were heated to 39°C for 20 min. The ability to perform on-chip isothermal heating was necessary for real-time observations of the entire PWA chip during RPA amplification.

### Wide-field fluorescence image acquisition and quantification

A wide-field, high-resolution imaging setup was required to adequately resolve all 27,000 picoliter wells. As illustrated in Fig 2a, large field of view images were captured using an Olympus microscope SZX7 (Olympus, Tokyo, Japan) and a monochrome cooled camera MC21 (Micro-shot Technology, Guangzhou, China) equipped with a 1.4 million pixel high-resolution CCD sensor (SONY, Tokyo, Japan). A resolution of 16 pixels per microwell can be achieved for the whole PWA chip. These were not sufficient to generate greater than 20 mW cm^-2^ light intensity on the entire chip area, which is necessary to excite the fluorescence signal for detection by the CCD camera. To address this problem, an optical cube was placed between the chip and object lens to shorten the path of excitation light to reach the fluorescent material. This improvement could reduce the loss of light intensity using a conventional microscope. The cube filters (Chroma Technology Corp., Bellows Fall, VT, USA) were composed of a 505-nm dichroscope, a filter set of 480/30 nm for fluorescence excitation and 535/40 nm for emission collection of the FAM dye, and were positioned 10 mm above the chip packaging device. The light source, a high-power 3 W blue LED bead (Micro-shot Technology, Guangzhou, China), was coupled to the cube in a horizontal direction.

ImageJ (NIH, Bethesda, MD, USA) and custom Matlab code were used to systematically detect and quantify fluorescent “positive” microwells and to analyze their density and averaged fluorescence intensity [35]. Background subtractions and contrast enhancement were performed for quantification of the dRPA results. The image acquired at the beginning (0 min) was regarded as the background noise of the experiment; other images acquired subsequently subtract it in situ for contrast enhancement. To enhance the visual contrast, grayscale images were converted to green. The quantitative results were then compared to the expected number of positive microwells predicted from Poisson statistics for serial dilutions of the known sample concentration.

## Results and Discussion

### Silanization of the PWA chip to avoid cross-contamination

The manufactured finished PWA chip was 20.8 mm × 16 mm, with 27,000 closely packed microwells. The height and diameter of the completed wells were 40 μm and 100 μm, respectively, with edge-to-edge spacing of 20 μm and volumes of 314 pL. A scanning electron microscope (SEM) image of the silicon picoliter well array is shown in Fig 2b. Native silicon is an inhibitor of PCR [70]; accordingly, the chip surface was coated with a compact 200-nm-thick silicon dioxide (SiO_2_) layer by thermal oxidation. However, the chip surface still absorbed polymerase protein and other reaction reagents, primarily owing to the great increase in the surface-to-volume ratio in the micro-scale environment, thus decreasing the DNA amplification efficiency. The conventional strategy involves treating the reaction chambers with 0.2% bovine serum albumin (BSA) solution at 80°C for 30 min before loading the PCR reaction mix [71] or adding 1 μL of 50 mg mL^−1^ BSA solution to 20 μL of PCR master mixture as a blocking protein to occupy the adsorption sites and reduce the loss of polymerase [9].

**Fig 3:**
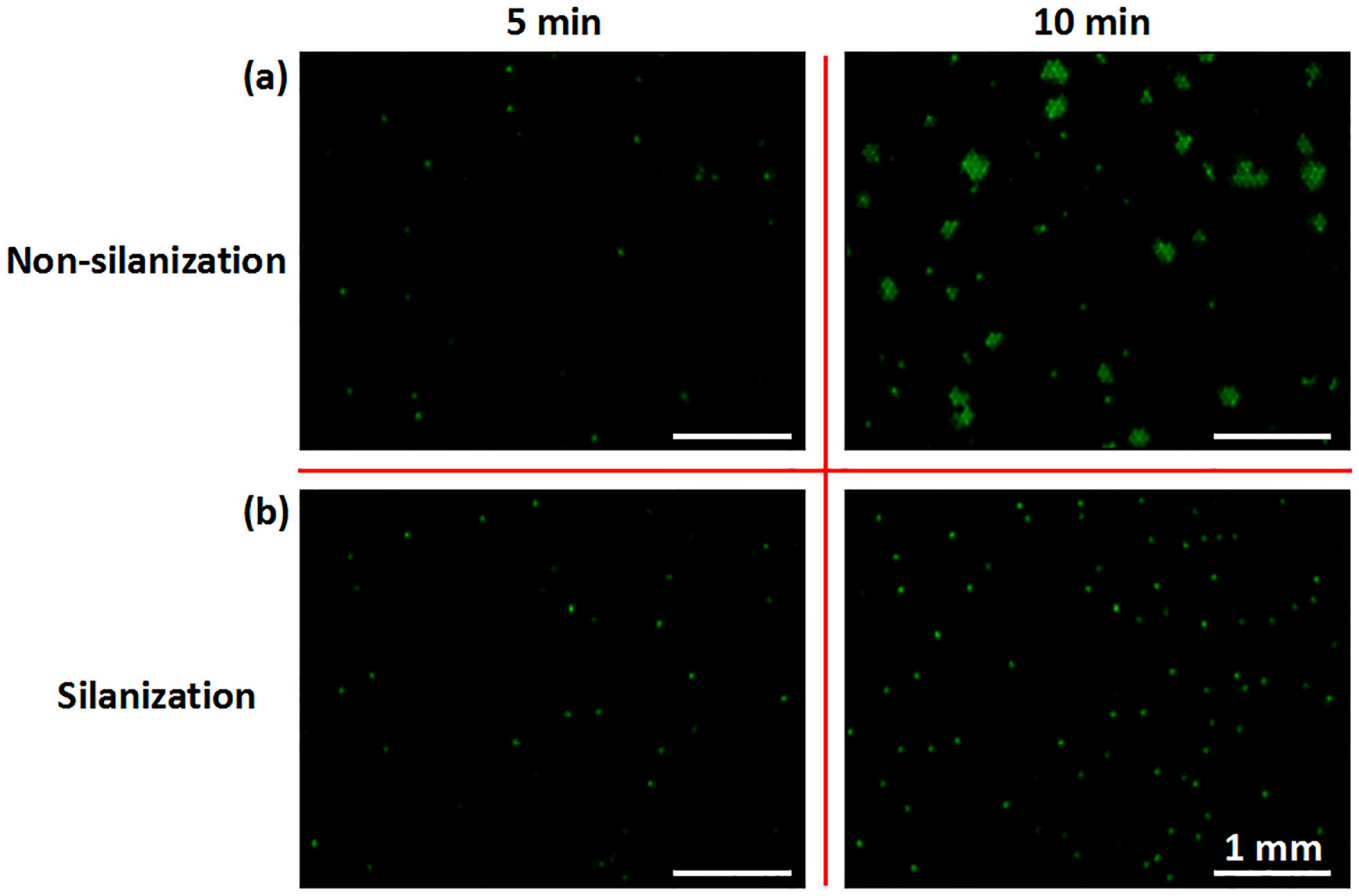
Digital RPA on the non-silanization and silanization chip. Real-time images of RPA amplification at 5 min and 10 min are shown. For a gDNA sample concentration of 0.53 pg μL^-1^, there are 1.2 copies of gDNA per 100 wells, on average. Scale bar represents 1 mm. (a) Many clusters of positive RPA wells appeared gradually from 5 min to 10 min, with cross-contamination on the non-silanization chip. (b) Positive RPA wells amplified independently from 5 min to 10 min, without cross-contamination on the silanized chip.

The “BSA strategy” can similarly ensure the success of RPA reactions in the picoliter wells, but it cannot eliminate cross-contamination that may occur between adjacent microwells owing to the edge-to-edge spacing of only 20 μm. There may be a thin liquid film on the chip surface after scraping PRA reagents into the picoliter wells. Although it will evaporate quickly, i.e., in 10–30 s, before oil sealing, the precise evaporation time is unknown because it is related to environmental humidity and temperature, which vary among experiments. The DNA amplification products enter adjacent microwells by residual liquid passage; thus, many clusters of positive RPA wells appear gradually from 5 min to 10 min with cross-contamination on the non-silanization chip (Fig 3a). Passivating the PWA chip surface by a silanizing agent may effectively eliminate cross-contamination during dRPA. Methoxy-PEG-silane could produce a compact monomolecular layer on the SiO_2_ surface by covalent binding (Fig 2c), which can protect PCR enzymes from being adsorbed and does not interfere with PCR reactions [72]. Additionally, it is highly compatible with mineral oil for sealing and fixes water molecules by hydrogen bond interactions. Accordingly, even when minor residual liquid remains after evaporation for 20 s, it is unable to flow on the methoxy-PEG-silane surface and connect adjacent microwells. There is no cross-contamination on the silanization chip; positive wells with a single DNA template amplify independently and the fluorescence intensity for adjacent negative wells does not change (Fig 3b). Moreover, the silanization of the PWA chip is very easy to perform, unlike the complicated modifications required for OpenArray, with a hydrophilic interior surface and hydrophobic exterior surface [72].

### Sample loading and chip packaging

We developed a simple and effective scraping liquid blade to partition a sample into as many as 27,000 independent reaction microwells. We scraped liquid with a certain strength to make the soft silica gel contact closely with the chip surface. Thus, after dispensing the reagent effectively, there was lesser residual liquid on the surface for reducing the opportunity of cross-contamination [73]. To produce a large number of monodispersed droplets, the scraping time (~5 s) is much shorter than that of other microdroplet techniques, in which droplets are commonly produced sequentially by T-junctions or flow [37–41]. Current digital droplet systems take considerable time for droplet production, which leads to amplification in the bulk solution. Thus, they cannot be used to perform dRPA. In contrast, the platform presented here can be applied to many DNA amplification techniques, such as RPA, PCR, RCA, and LAMP. This sample loading style does not require pumping equipment or precise microvalve control [36].

In the scraping process, a relatively slow operation is suitable to fill microwells with the liquid reagent; otherwise, the wells may contain air bubbles in the bottom. Once heated, the bubbles rush out, diffuse, and cause reaction failure on the PWA chip. In contrast to previously performed dRPA reactions on the SlipChip, the PWA chip works without precise “slip” control [66]. If the SlipChip reduces the microwell volume and increases the density rapidly for a high-throughput detection like the PWA chip, the complexity and precision of the “slip” operation would increase rapidly, too. In contrast to previously performed dRPA reactions in droplets produced by centrifugal step emulsification, the droplets of the PWA chip are more consistently sized, avoiding the uneven droplets volumes affecting the quantification results, and their volumes are smaller, i.e. picoliter-sized [67]. Reagent consumption for each experiment on the PWA chip was less than 20 μL. A small reaction volume not only contributes to powerful and high-throughput detection, but also generates much less reagent waste and reduces the cost of each reaction.

Chip packaging to seal the PWA is another key technology. The picoliter droplets evaporate easily in the incubation, even when sealed with mineral oil. When the reagent concentration increases to a certain degree owing to water evaporation, the RPA reaction is not carried out in the picoliter wells, and the more demanding dRPA cannot be performed. Thus, we designed a chip chamber to seal the finished PWA chip after sample loading. The glass cover-plate serves to create a vapor barrier to reduce oil and RPA reagents from evaporating during incubation [9]; and the rubber O-ring strengthens the air tightness between the glass and the copper chamber when the screws are tightened at the four corners. Conventional dPCR can be also performed successfully on the PWA chip with the packaging device, for a thermocycling temperature and reaction time of up to 95°C and 2 h, respectively (unpublished results).

### Performance of real-time dRPA on the PWA chip

As an isothermal amplification technique, RPA cannot be simply triggered using a “hot-start,” which is established in conventional PCR. Instead, the RPA reaction mixture (i.e., the oligo mix, buffer, template, and enzymes) is prepared without the addition of Mg^2+^ to prevent the reaction from starting. After Mg^2+^ is added, the reaction proceeds, sometimes at room temperature. To disable amplification between the addition of the MgAc solution to the RPA reaction mixture and sample loading, reagents were pre-cooled to 0°C during preparation. After the MgAc solution was added to the RPA reaction mixture, the sample loading operation was completed in less than 15 s, allowing very little time for the solution to heat or for amplification to begin. Additionally, no thermal cycling is necessary for RPA, thus enabling very easy and cheap digital amplification.

**Fig 4:**
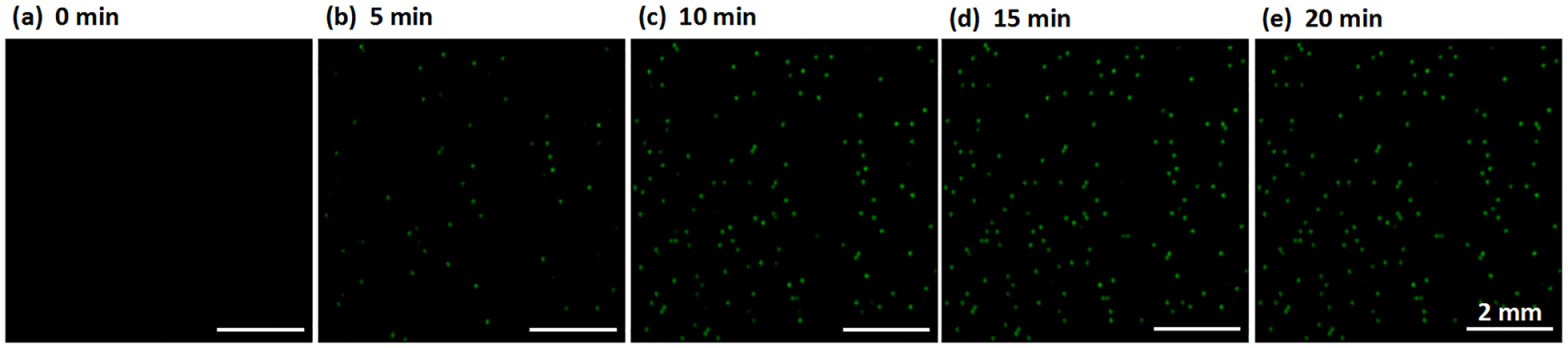
Real-time imaging of RPA amplification for 0–20 min. Scale bar represents 2 mm.

We modified a commercial fluorescence microscope to minimize the distance between the optical cube and the PWA chip, which ensures sufficient light intensity to fully excite the fluorescence signal to obtain wide-field, high-resolution images. This method affords several significant advantages over previous approaches: (a) The macro-fluorescence imaging setup is capable of viewing 6-cm^2^ areas in a single snapshot at 0.8× magnification, without the need to obtain images by scan shooting and stitching serial small images together [34]. Thus, image acquisition and processing are rapid. (b) This approach allows the excitation light to vertically illuminate the chip through the optical cube and avoids flatfield corrections due to oblique beam illumination [74]. An uneven light field affects the quantification results. (c) The wide-field images are captured in situ, enabling real-time fluorescence detection. Real-time fluorescence imaging of RPA amplification is demonstrated in Fig 4 for a 20-min period at 5-min increments (these images are enlarged sections from the wide-field fluorescence images for enhanced visualization).

Instead of monitoring only the end-point fluorescent intensity, which is done in many single DNA molecular application experiments, our real-time fluorescence detector is useful for investigating amplification uniformity and for optimizing the total time required for incubation [74]. As shown in Fig 4, the RPA amplification is rapid, such that the number of positive points no longer increases after 15 min. Overall dRPA-on-chip processing requires less than 30 min, which is a 4-fold decrease compared to dPCR, with a processing time of approximately 2 h. The reduced time to results is especially important for fast field analysis and critical clinical applications, such as in cases of sepsis. Furthermore, real-time analysis improves detection sensitivity because it allows the use of a reference frame to subtract background noise, which cannot be achieved with a single end-point frame. Temporal information also helps to differentiate between amplified signals and noise because the rate at which the intensity changes can be expected to increase gradually from frame to frame, providing another parameter by which to detect positive droplets. A plot of fluorescence intensity vs. reaction time is shown in S3 Fig.

**Fig 5:**
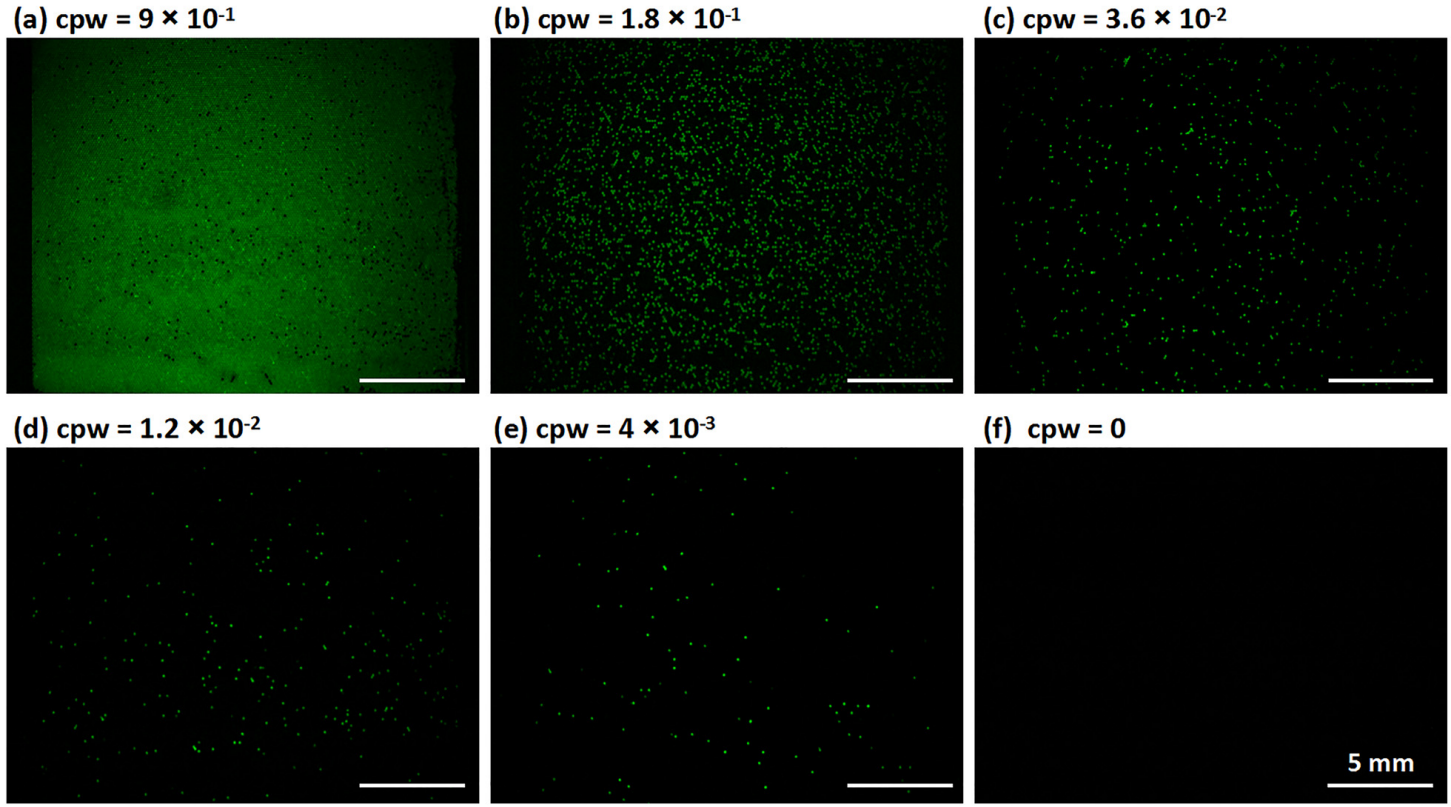
Digital RPA on the PWA chip for different concentrations of *Listeria monocytogenes* gDNA. (a—e) Digital RPA on the PWA chip with serial dilutions of target DNA template ranging from 9 × 10^-1^ to 4 × 10^-3^ expected copies per well (cpw). (f) Control, no wells showed positive signals when no target DNA was loaded.

**Fig 6:**
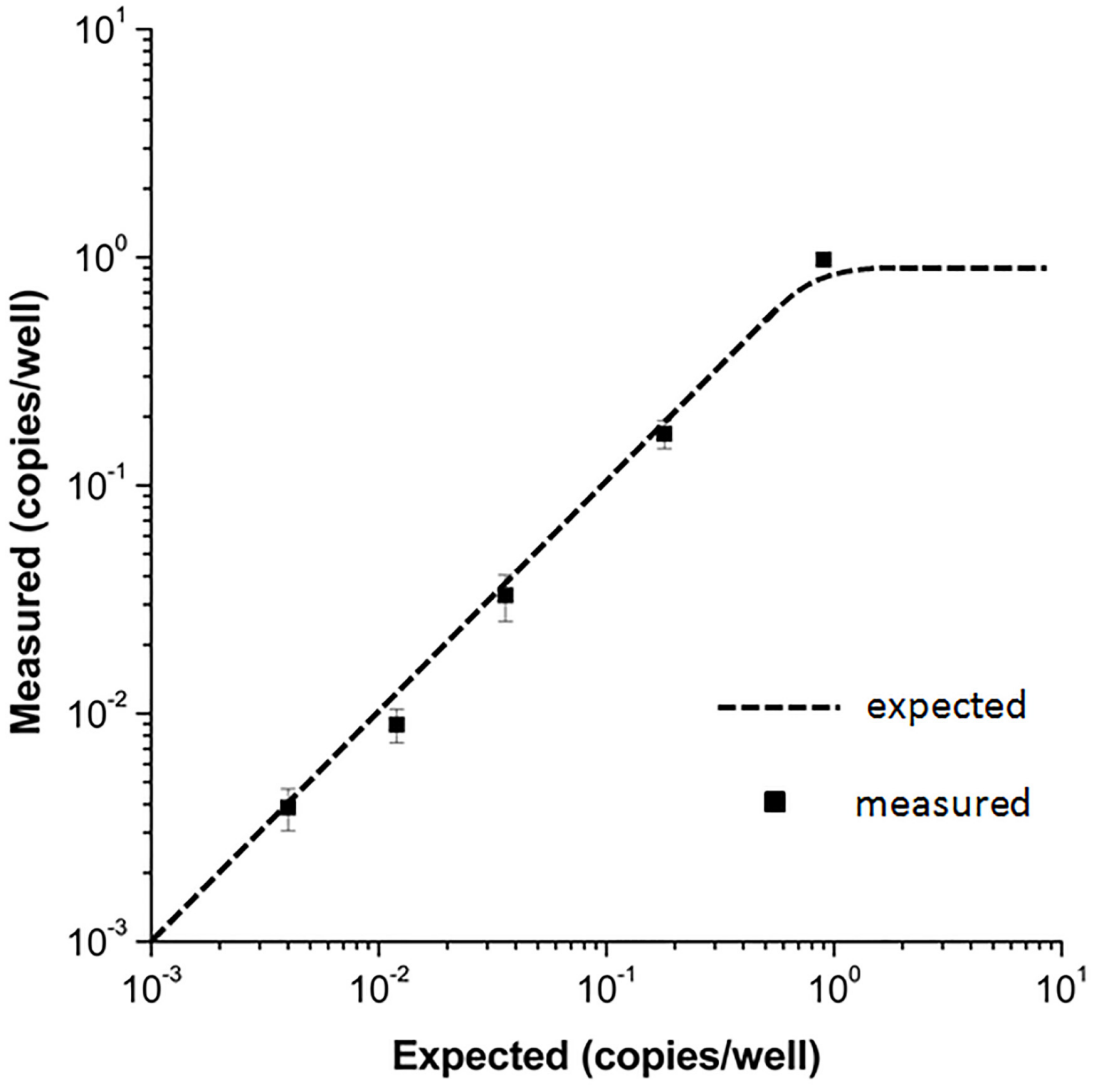
Quantitative results of digital RPA on the PWA chip. The measured copies per well (cpw) was highly concordant with the expected cpw, with an average error rate of less than 11% (N = 15). Error bars represent standard deviations.

We characterized the performance of the PWA chip for digital RPA using serial dilutions of a *L*. *monocytogenes* gDNA stock solution (Fig 5). The expected concentration of the gDNA template was estimated as the number of copies per well (cpw), and the concentration of the original gDNA stock solution was verified spectrophotometrically. The detailed method for calculating the expected cpw is presented in the Preparation of dRPA reagents section. As the gDNA template was diluted, the expected cpw values were 9 × 10^-1^, 1.8 × 10^-1^, 3.6 × 10^-2^, 1.2 × 10^-2^, and 4 × 10^-3^. After incubation, the number of positive RPA wells on the PWA chip decreased proportionally (Fig 5a–5e). No evidence of contamination was observed as no false positives were observed in the negative control (Fig 5f). We repeated the experiments three times at each gDNA concentration to test the robustness and reproducibility of digital RPA on the PWA chip. A Poisson statistical analysis of the dRPA results was performed as previously described [35,66]. As shown in Fig 6, the measured cpw with dRPA was highly concordant with the expected cpw, with an average error rate of less than 11% (N = 15). Accurate qPCR experiments can estimate original DNA concentrations with variation of less than 1 cycle number, resulting in 150–200% errors in original concentration estimates [75]; accordingly, this dRPA method had better performance. Furthermore, it is noteworthy that potential pipetting errors during dilution and potential losses of DNA during sample preparation could increase the deviation between two estimates.

## Conclusions

We developed a PWA chip with 27,000 consistently sized wells (314 pL) to perform isothermal DNA quantification using dRPA at 39°C for 20 min on a homemade isothermal heater. Sample loading using a scraping liquid blade was simple, fast, and consumed minimal reagents (< 20 μL). Passivating the PWA chip surface by a methoxy-PEG-silane agent eliminated cross-contamination among wells. Our optical design enabled wide-field fluorescence imaging in situ, with both end-point and real-time analysis of picoliter wells in 6-cm^2^ areas during dRPA. We applied the PWA chip to accurately quantify serial dilutions of a *L*. *monocytogenes* gDNA stock solution. The dRPA reaction was robust and free of cross-contamination on the PWA chip, but the specificity of the device and strategies for multiplex detection remain to be examined. The digital PWA chip can be readily applied to other nucleic acid amplification techniques, such as PCR, RCA, ELISA, and LAMP.

## Supporting Information

S1 Fig Calculating method of DNA copies per well. (TIF)

S2 Fig (a) Bright-field image of the sample loading instrument containing a chip carrier and a scraping liquid blade, displayed on a ruler to show scale. The scraping liquid blade is composed of a glass slide and a piece of silica gel (thickness, 3 mm), which are pasted together at an end; the chip carrier is composed of another glass slide and a 3M adhesive tape, which prevents the chip from sliding in the process of scraping. (b) After sample loading by scraping the RPA reagents into the picoliter wells array, let the PWA chip sit quietly in room temperature for 20 seconds for the little residual liquid evaporating. Then sealing the chip with excess mineral oil via a disposable pipette until the entire surface was fully covered. (c-e) Transferring the sample loading finished PWA chip from the chip carrier to the copper chamber filled with mineral oil. (f) Fixing the glass cover-plate on the copper chamber with screws. The rubber O-ring is added around the chamber to strengthen the air tightness. The whole process avoids air bubbles.
(TIF)

S3 Fig A plot of relative fluorescence intensity vs. reaction time within 20 positive picoliter wells and 20 negative picoliter wells. The acquisition time was 0 min, 2.5 min, 5 min, 7.5 min, 10 min, 12.5 min, 15 min, 17.5 min, 20 min. The fluorescence intensity of some RPA positive points increases rapidly in a short time.
(TIF)

S1 File The Poison statistical analysis of dRPA results. (PDF)

S1 Table The sequences of the primers and probe for dRPA-on-chip (all in 5′→ 3′direction). (PDF)
