# Supplementary figures and images for: Picoliter Well Array Chip-Based Digital Recombinase Polymerase Amplification for Absolute Quantification of Nucleic Acids
Source: PLoS One. 2016 Apr 13;11(4):e0153359. doi: 10.1371/journal.pone.0153359 (PMC4830604; doi:10.1371/journal.pone.0153359)

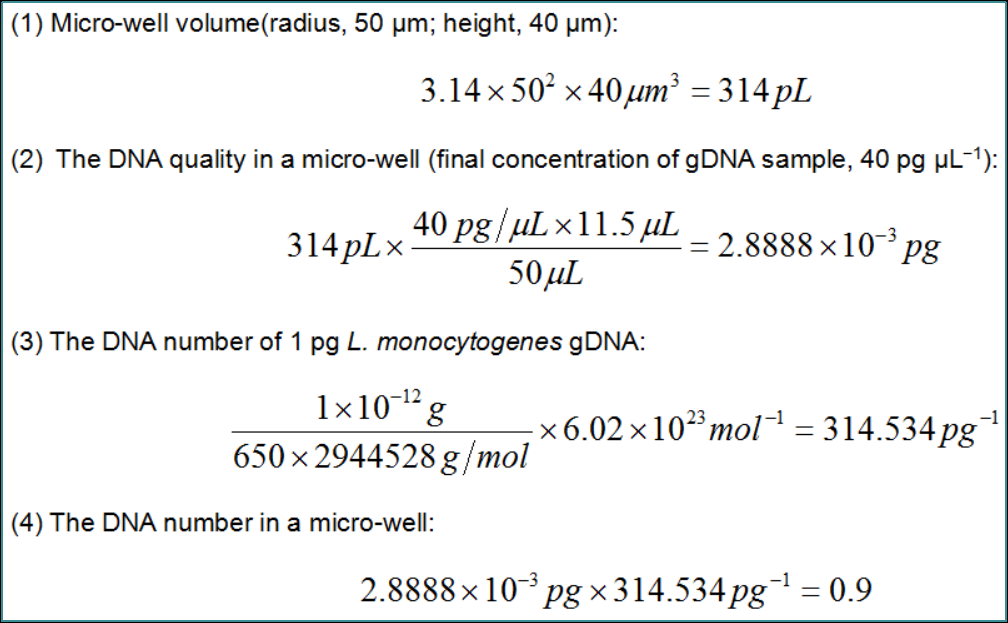

Supplement: S1 Fig — (TIF) [file pone.0153359.s001.tif]

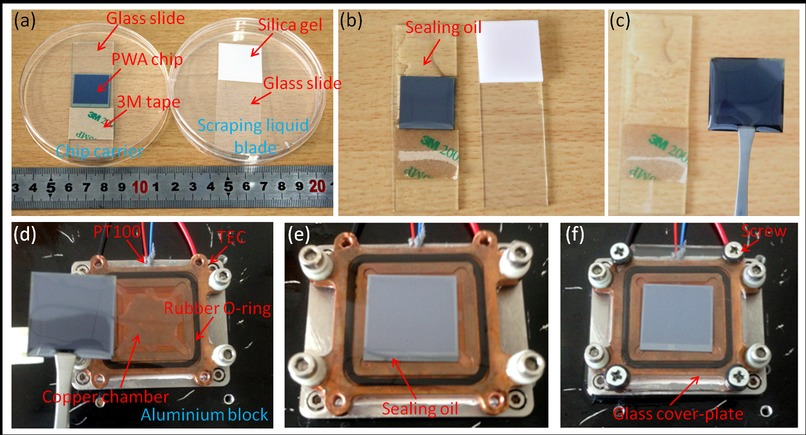

Supplement: S2 Fig — (a) Bright-field image of the sample loading instrument containing a chip carrier and a scraping liquid blade, displayed on a ruler to show scale. The scraping liquid blade is composed of a glass slide and a piece of silica gel (thickness, 3 mm), which are pasted together at an end; the chip carrier is composed of another glass slide and a 3M adhesive tape, which prevents the chip from sliding in the process of scraping. (b) After sample loading by scraping the RPA reagents into the picoliter wells array, let the PWA chip sit quietly in room temperature for 20 seconds for the little residual liquid evaporating. Then sealing the chip with excess mineral oil via a disposable pipette until the entire surface was fully covered. (c-e) Transferring the sample loading finished PWA chip from the chip carrier to the copper chamber filled with mineral oil. (f) Fixing the glass cover-plate on the copper chamber with screws. The rubber O-ring is added around the chamber to strengthen the air tightness. The whole process avoids air bubbles. (TIF) [file pone.0153359.s002.tif]

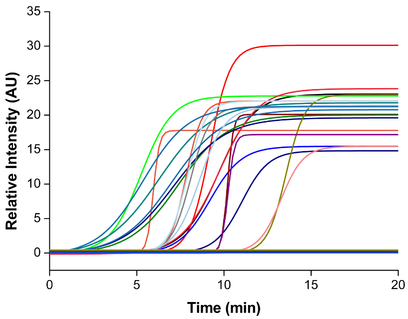

Supplement: S3 Fig — The acquisition time was 0 min, 2.5 min, 5 min, 7.5 min, 10 min, 12.5 min, 15 min, 17.5 min, 20 min. The fluorescence intensity of some RPA positive points increases rapidly in a short time. (TIF) [file pone.0153359.s003.tif]
