# Supplementary material for: Picoliter Well Array Chip-Based Digital Recombinase Polymerase Amplification for Absolute Quantification of Nucleic Acids
Source: PLoS One. 2016 Apr 13;11(4):e0153359. doi: 10.1371/journal.pone.0153359 (PMC4830604; doi:10.1371/journal.pone.0153359)
Supplement: S1 File — (PDF) [file pone.0153359.s004.pdf]

S1 File. The Poison statistical analysis of dRPA results.

The Poisson equation:

$$p = (\lambda^k \bullet e^{-\lambda}) / k! \quad k = 0, 1, 2, 3, ...$$

where p is the probability of having k templates in a well given λ. λ is the average number of DNA templates per well. In the digital reaction, when the template number $k > 0$, the amplification reaction in the well will proceed, and its fluorescence intensity will increase, which is regarded as "positive" well; when the template number $k = 0$, there is no amplification reaction in the well, and its fluorescence intensity will not increase, which is regarded as "negative" well.

The equation simplifies to $\lambda = -\ln(1-p)$ when $k > 0$. We can get the "measured" number of copies per well (cpw) λ_m by using poison statistics, where $p = f/n$. f is the number of positive wells, detected by the optical setup through analyzing the increasing fluorescence intensity. n is the total number of wells on the chip.

The "expected" number of cpw is $\lambda_e = c_0 \bullet v \bullet x_{dil}$, and c_0 is the stock concentration of DNA templates, v is the volume of each chamber, and x_{dil} is the dilution factor. Therefore, we can assess the performance of dRPA method by comparing the correlation between the "measured" cpw and the "expected" cpw.
