# Supplementary material for: Picoliter Well Array Chip-Based Digital Recombinase Polymerase Amplification for Absolute Quantification of Nucleic Acids
Source: PLoS One. 2016 Apr 13;11(4):e0153359. doi: 10.1371/journal.pone.0153359 (PMC4830604; doi:10.1371/journal.pone.0153359)
Supplement: S1 Table — (PDF) [file pone.0153359.s005.pdf]

Supporting information

Picoliter well array chip-based digital recombinase polymerase amplification for absolute quantification of nucleic acids

Zhao Li[1], Yong Liu[1], Qingquan Wei[1], Yuanjie Liu[1], Wenwen Liu[1], Xuelian Zhang[1], Yude Yu[1,2*]

[1]State Key Laboratory on Integrated Optoelectronics, Institute of Semiconductors, Chinese Academy of Sciences, P.O. Box 912, Beijing, 100083, China. E-mail: yudeyu@semi.ac.cn; Fax: +86 10 82305052; Tel: +86 10 82304979

[2]Joint Laboratory of Bioinformation Acquisition and Sensing Technology, Institute of Semiconductors, Beijing Institute of Genomics, Chinese Academy of Sciences, P.O. Box 912, Beijing 100083, China.

S1 Table. The sequences of the primers and probe for dRPA-on-chip (all in 5′→ 3′direction).

Forward primer	CGCCTGCAAGTCCTAAGACGCCAATCGAAAAGAAAC
Reverse primer	CTGCATCTCCGTGGTATACTAATACATTGTTTTTA
Probe	CGAAAAGAAACACGCGGATGAAATCGATAAG[FAM][THF][BHQ-1]ATACAAGGATTGGA

S1 Fig. Calculating method of DNA copies per well.

(1) Micro-well volume(radius, 50 μm; height, 40 μm):

$$3.14 \times 50^2 \times 40 \mu m^3 = 314 pL$$

(2) The DNA quality in a micro-well (final concentration of gDNA sample, 40 pg μL^{-1}):

$$314 pL \times \frac{40\, pg/\mu L \times 11.5\, \mu L}{50 \mu L} = 2.8888 \times 10^{-3}\, pg$$

(3) The DNA number of 1 pg *L. monocytogenes* gDNA:

$$\frac{1 \times 10^{-12} g}{650 \times 2944528\, g/mol} \times 6.02 \times 10^{23} mol^{-1} = 314.534 pg^{-1}$$

(4) The DNA number in a micro-well:

$$2.8888 \times 10^{-3} pg \times 314.534 pg^{-1} = 0.9$$

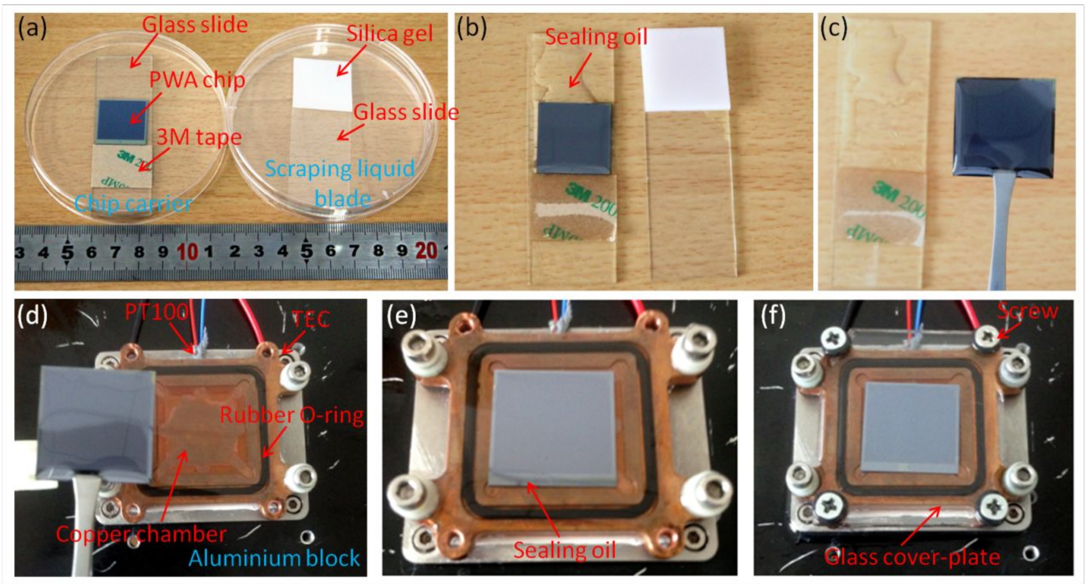

S2 Fig. (a) Bright-field image of the sample loading instrument containing a chip carrier and a scraping liquid blade, displayed on a ruler to show scale. The scraping liquid blade is composed of a glass slide and a piece of silica gel (thickness, 3 mm), which are pasted together at an end; the chip carrier is composed of another glass slide and a 3M adhesive tape, which prevents the chip from sliding in the process of scraping. (b) After sample loading by scraping the RPA reagents into the picoliter wells array, let the PWA chip sit quietly in room temperature for 20 seconds for the little residual liquid evaporating. Then sealing the chip with excess mineral oil via a disposable pipette until the entire surface was fully covered. (c-e) Transferring the sample loading finished PWA chip from the chip carrier to the copper chamber filled with mineral oil. (f) Fixing the glass cover-plate on the copper chamber with screws. The rubber O-ring is added around the chamber to strengthen the air tightness. The whole process avoids air bubbles.

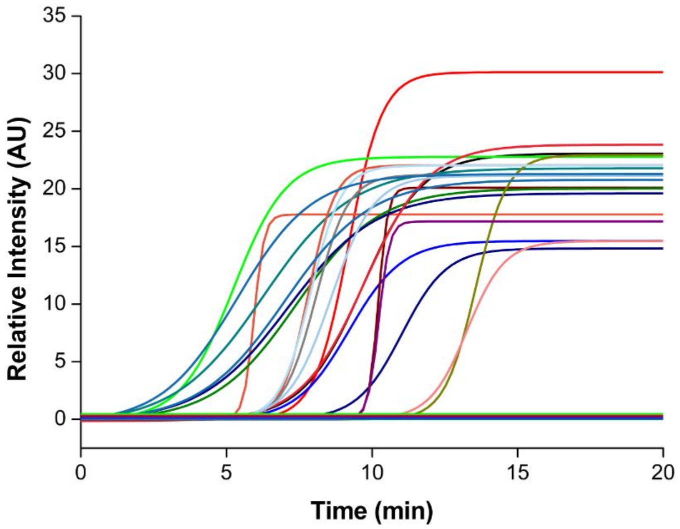

S3 Fig. A plot of relative fluorescence intensity vs. reaction time within 20 positive picoliter wells and 20 negative picoliter wells. The acquisition time was 0 min, 2.5 min, 5 min, 7.5 min, 10 min, 12.5 min, 15 min, 17.5 min, 20 min. The fluorescence intensity of some RPA positive points increases rapidly in a short time.

S1 File. The Poison statistical analysis of dRPA results.

The Poisson equation:

$$p = (\lambda^k \bullet e^{-\lambda}) / k! \quad k = 0, 1, 2, 3, ...$$

where p is the probability of having k templates in a well given λ. λ is the average number of DNA templates per well. In the digital reaction, when the template number $k > 0$, the amplification reaction in the well will proceed, and its fluorescence intensity will increase, which is regarded as "positive" well; when the template number $k = 0$, there is no amplification reaction in the well, and its fluorescence intensity will not increase, which is regarded as "negative" well.

The equation simplifies to $\lambda = -\ln(1-p)$ when $k > 0$. We can get the "measured" number of copies per well (cpw) λ_m by using poison statistics, where $p = f/n$. f is the number of positive wells, detected by the optical setup through analyzing the increasing fluorescence intensity. n is the total number of wells on the chip.

The "expected" number of cpw is $\lambda_e = c_0 \bullet v \bullet x_{dil}$, and c_0 is the stock concentration of DNA templates, v is the volume of each chamber, and x_{dil} is the dilution factor. Therefore, we can assess the performance of dRPA method by comparing the correlation between the "measured" cpw and the "expected" cpw.
